# Multi-level modelling of the factors that influence the participation of disabled rural individuals in social medical insurance in China

**DOI:** 10.1186/1472-6963-13-58

**Published:** 2013-02-12

**Authors:** Ju Huang, Xi-Long Pan, Ang Li

**Affiliations:** 1 Department of Health Policy and Management, School of Public Health, Peking University Health Science Center, 38 Xueyuan Road, Beijing, Haidian District, China

**Keywords:** Multilevel modeling, Influencing factor, Rural disabled persons, Social medical insurance

## Abstract

**Background:**

The Second China National Sample Survey on Disability in 2006 showed that the participation rate of disabled Chinese rural individuals in social medical insurance participation was less than 30%. However, there has been limited number of studies on the influencing factors, especially contextual factors, affecting their participation in social medical insurance. Therefore, this study aimed to analyze the factors influencing the participation of disabled rural individuals in social medical insurance, including contextual factors.

**Methods:**

Based on data derived from the Second China National Sample Survey on Disability, chi-square test and two-level logistic regression model were used to analyze the influencing factors.

**Results:**

The results showed that the disabled rural individuals in the New Rural Cooperative Medical System pilot counties who lived in communities with rehabilitation stations or with higher per capita income of villagers were more likely to participate in the social medical insurance. Meanwhile, those employed, with less severe disability degree or with less severe barriers in participation in society were more likely to participate in the social medical insurance.

**Conclusions:**

Contextual factors including economic and policy contexts were important factors influencing their participation in social medical insurance before 2006 in China. Unemployment, severer disability degree and social isolation might also prevent them from gaining equal access to social medical insurance.

## Background

Disability^(a)^ is a serious issue in the whole world drawing increasing attention. Some international organizations and countries have introduced programs or laws to protect their rights. World Program of Action Concerning Disabled Persons [1] approved by the United Nations in 1982 and Convention of the United Nations on the Rights of Persons with Disabilities [2] passed in 2006 stressed on the full participation and equal rights of the disabled persons. In China, the Law of the People’s Republic of China on the Protection of Disabled Persons [3] enacted in 1990 and was revised in 2008. These are the current laws protecting their legal rights in China. In spite of this, the medical security of disabled persons was largely restrained, thus the new Medical Reform of China [4] emphasized on their medical security.

There are 650 million disabled individuals all over the world, accounting for 10% of the world’s total population [5]. According to the Second China National Sample Survey on Disability (SCNSSD), by April 1, 2006, the total number of the disabled in China was 82.96 million, accounting for 6% of the national population. Among them, the majority (62.25 million, 75% of all the disabled population) were in the rural areas [6]. The total number of households with disabled persons was 70.50 million, accounting for 18% of the national households [6]. However, despite the large population of this vulnerable group, only 32% of them were covered by social medical insurance schemes in 2006 [7] and the coverage was generally lower in the rural areas (average of 28%) as compared to the urban areas. The coverage also varied in different provinces ranging from under 10% to 79% [8,9].

Disabilities may cause diseases that might lead to poverty and poverty might in return cause diseases that reinforce disabilities. Therefore, a vicious circle shadows the life of some disabled individuals [10]. In fact, 42% of the absolutely poverty-stricken people are disabled [11]. The results from the Sample Survey on one thousandth of disabled individuals in Beijing in 2005 showed that the average expenditure on medical rehabilitation of the disabled individuals was 4,108 Yuan, accounting for 81% of their average annual income, but 176% of the annual income of the disabled in rural areas [12]. The survey also revealed that 60-70% of the rural disabled received no medical rehabilitation [13].

Studies on social medical insurance of the disabled are quite few in China, especially before 2006 when the lack of survey data made it impossible to carry out comprehensive studies on social medical insurance of disabled individuals. Based on the data of the SCNSSD, some domestic scholars carried out studies on medical security [14-18]. However, these studies had certain limitations on the theory and method design. Yintong [17] reported that the policy context might affect the disabled persons’ social medical insurance participation. However, due to the inclusion of only urban disabled persons and the limitations mentioned above, it was difficult to determine the influence of policy context on disabled persons’ participation in social medical insurance. Zhoumi [18] tried to explore the factors, which might influence disabled persons’ purchase of social medical insurance; however, it was just restricted to one province. Thus, whether policy context or other factors have influence on rural disabled persons’ participation in social medical insurance is unknown. At the same time, scholars have focused their attention on the barriers caused by disability rather than by the social medical insurance system and the policy context. This study utilized scientific and practical methods to explore the influences on rural disabled persons’ participation in social medical insurance. We hypothesized that contextual factors, including policy and economic factors could influence rural disabled persons’ participation in social medical insurance. Furthermore, we hypothesized that with the increasing severity of disability degree, contextual factors might have increasing influences on rural disabled persons’ participation in social medical insurance.

## Methods

### Setting and sampling

This study was based on the data derived from SCNSSD. In order to understand the latest situation of the disabled individuals so as to help formulate development plans and relevant regulations, China carried out the SCNSSD in 2006. There are many definitions for “disabled person/person with disability”. The SCNSSD and this study adopted the concept of disabled persons in Law of the People’s Republic of China on the Protection of Disabled Persons. Based on these, the disabled person refers to people who have lost all or part of the ability to engage in certain activities in the normal way as a result of certain tissue or function loss or psychological, physiological or anatomical abnormality. This definition also includes people with visual, hearing, speech, physical and mental disabilities along with mental retardation or multiple disabilities [3].

**Figure 1:**
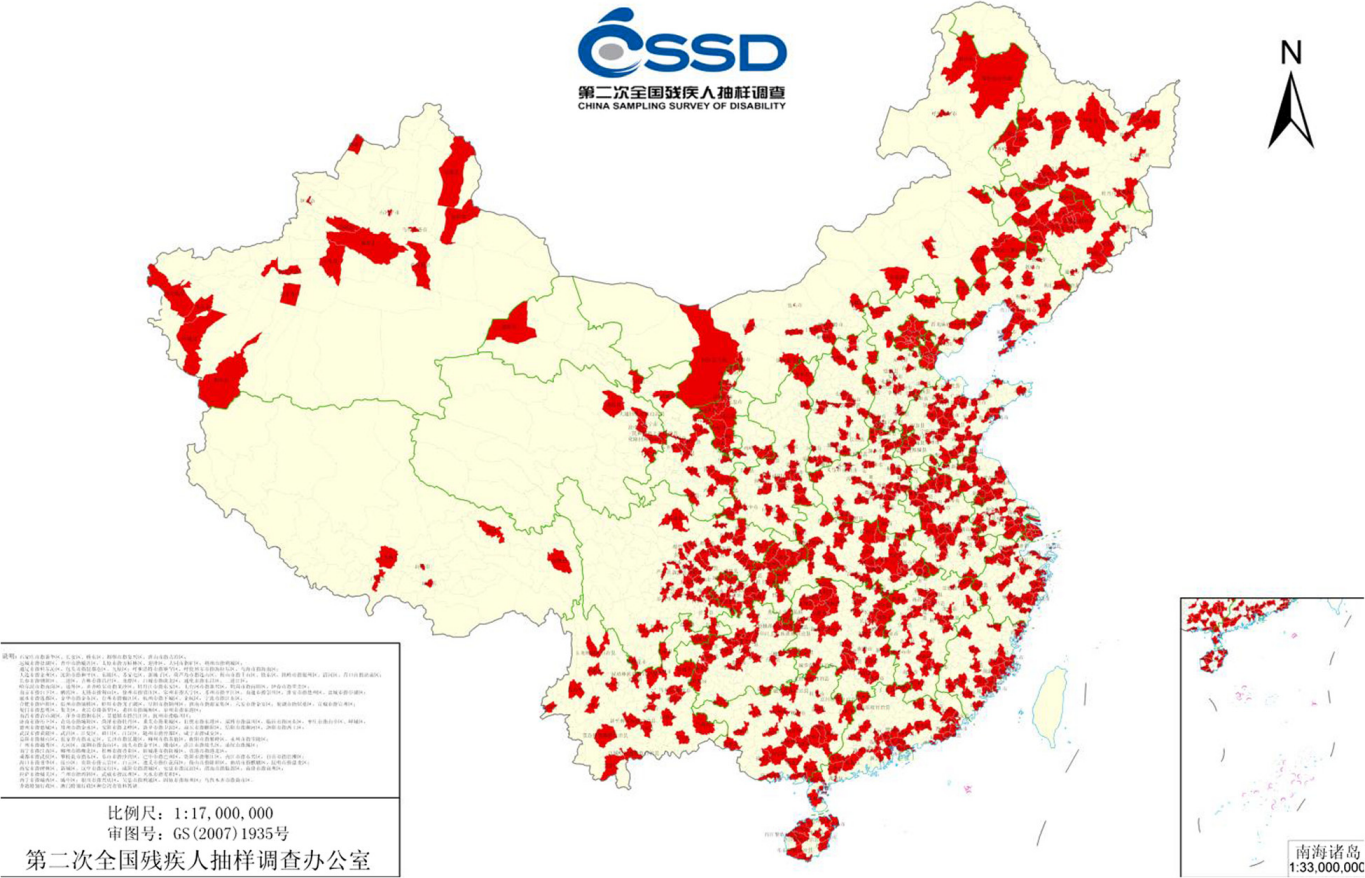
Distribution of sampled counties by geography.

The SCNSSD was a population-based, nationally representative survey that was conducted in 2006 in China. The target population was households that were sampled from 31 provinces, autonomous regions and municipalities in China (Figure 1). A stratified, multiphase and cluster probability sampling design was used to randomly sample participants at four levels including county, town, village and community. The sample size in each level was based on the proportion of population to the province. Four towns were selected in each county, two villages in each town and one community in each village. Finally, the SCNSSD sampled a total of about 2,500,000 people from 5,964 communities of 2,980 towns in 734 counties.

This study was based on a part of the results from the SCNSSD database that was provided by China Disabled Persons Federation. According to the objective of this study, disabled persons above the age of 18 in Chinese rural areas were included. A total of 114,485 individuals were studied. This study was funded by China Disabled Persons’ Federation (2010&YB004).

### Data collection

In order to get the actual number of households and population, the investigators entered into the survey communities ahead of time. The “Survey Reference Book” was formulated based on the actual situation. On-site registration consisted of household survey and disability assessment. As for the household survey, the households were investigated under the guidance of “Survey Reference Book” using “Survey Questionnaire for Household”. As for the disability assessment, doctors of various specialties combined the stationary and household surveys to assess the disability on an individual basis. Finally, the completed survey questionnaires were reviewed comprehensively by investigators and doctors.

### Quality of data

#### Representativeness

The comparison of the age structure, sex ratio and other demographic indicators of the SCNSSD data in 2006 with the data from the 1% Population Sample Survey in 2005 showed that the sample had a good representativeness.

#### Integrity

The post-survey quality check showed that the omission rate was 1.31% for the registered population and 1.12% for the disabled population. This was an indication of a high integrity.

#### Reliability

Under the 95% and 90% confidence levels, the allowable errors for the proportion of surveyed disabled population to the national population were 0.97‰ and 0.80‰ respectively. According to some experimental data provided by the United Nation (UN) experts, the data with a relative error of less than 10% was considered reliable. Based on this, the SCNSSD data was reliable.

#### Accuracy

The Whipple’s index was 96.00 for the males and 96.10 for the females, which were very close to 100. Furthermore, the Myer’s index was 1.55, which was less than 5, indicating that the SCNSSD data was accurate.

In summary, the SCNSSD data had a high quality.

### Variables description

#### Outcome measure (Dependent variable)

The dependent variable was Participation in Social Medical Insurance, which was binary (1 = Yes; 0 = No).

#### Individual-level variables (Independent variables)

The individual-level variables included demographic indicators (gender, age, educational level and marital status), economic status indicator (Per Capita Household Income (PCHI)) and disability status indicators (disability type, disability degree and participation in society).

Gender was a dummy variable, coded as 1 and 2, representing male and female, respectively. Age was a continuous variable, representing participant’s age at the time of the baseline interview. Educational level was a dummy variable, coded as 1, 2 and 3, representing college or above, primary, high and technical secondary school and primary school and below, respectively. Marital status was a dummy variable, coded as 1 and 2, representing in marriage and not in marriage, respectively.

Employment was a dummy variable, coded as 1 and 2, representing employed and unemployed, respectively.

PCHI was based on the information gathered from the 1% of the census (2005), which indicated a poverty level of 683 Yuan [19]. The average per capita household income in our sample was 2,281 Yuan. Therefore, our analysis used three income levels, coded as 1, 2 and 3, representing 683 or less, 684–2,300, and 2,301 or more, respectively.

Disability degree was a dummy variable, coded as 1, 2, 3 and 4, representing grade-1 (most severe), grade-2, grade-3 and grade-4 (least severe), respectively. Participation in society was a dummy variable, coded as 1, 2 and 3, representing none/mild barriers, moderate barriers and severe barriers, respectively.

#### Community-level variables (Independent variables)

Community-level variables were indicators of the communities where the disabled lived, including an economic status indicator (Per Capita Income of the Villagers in 2005 (PCIV)), a policy indicator (New Rural Cooperative Medical System Pilot County (NPC)) and other contextual indicators (Special Committees for the Disabled (SCD) and rehabilitation stations).

The average PCIV in our sample was 2,800 Yuan. Therefore, in our analysis we used three income levels, coded as 1, 2 and 3, representing 683 or less, 684–2,800 and 2,801 or more, respectively. NPC was a dummy variable, coded as 1 and 2, representing NPC and non-NPC, respectively. SCD was a dummy variable, coded as 1 and 2, representing with SCD and without SCD, respectively. Rehabilitation station was a dummy variable, coded as 1 and 2, representing with rehabilitation stations and without rehabilitation stations, respectively.

More than 2,300 Yuan, grade-4, severe barriers, more than 2,801 Yuan, non-NPC, without SCD and without rehabilitation station served as the reference.

### Statistical analysis

The methods of calculating frequencies and averages were used to describe the independent variables. The *x*^*2*^*-test* was used to examine the independent associations between rural disabled persons’ social medical insurance participation rate and each independent variable.

**Figure 2:**
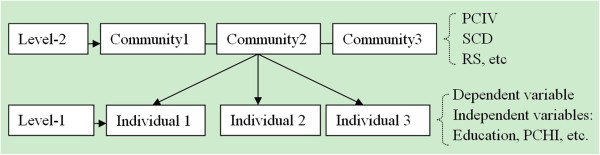
Hierarchical structure of the two-level model and distribution of relevant variables.

Because of the hierarchical structure of the data and the discrete outcome, multilevel logistic regression analysis, a type of the Generalized Linear Mixed Model [20], was used to assess the association between independent variables and rural disabled persons’ participation rate. Multilevel logistic regression model is an extension of fixed effect logistic regression, incorporating random effects into the model in order to deal with the intraclass correlation coefficient (ICC) that arises in multilevel data. This study established a Two-Level Logistic Regression Model, with the individual (including outcome measure and individual variables) as level-1 and the community as level-2. The hierarchical structure and variable distribution are shown in Figure 2.

In step-1, a null model was developed to assess the within-group homogeneity. The results of running an empty model showed a significant between-group variation in the mean outcome measure (ICC=0.8264, *σ*^2^*μ*_0_ = − 2.72, *p* < 0.0001). An ICC of 0.8264 was very large and indicated that 82.64% of the total variation in the mean outcome measure was caused by variation between the survey sites. The combined model was $\log\left(\frac{p_{\mathrm{ij}}}{1-p_{\mathrm{ij}}}\right)=\gamma_{00}+u_{0j}$, where *p*_*ij*_ was the probability of rural disabled persons’ participation in social medical insurance and *u*_0 *j*_ was the random variation in the level-1 intercept across groups (survey sites in this study), which represents the deviations of the *j*^*th*^ group’s mean logit from the overall mean logit.

As such, in step-2, contextual variables explaining the between-site variation are explored without adjusting for individual characteristics. Thus, the combined multilevel logit model was as follows:

$$\begin{array}{cc} \;\log\left(\frac{p_{\mathrm{ij}}}{1-p_{\mathrm{ij}}}\right) & =\gamma_{00}+\gamma_{01}\mathrm{PCIV}+\gamma_{02}\mathrm{NPC}+\gamma_{03}\mathrm{SCD}\\ & +\gamma_{04}\mathrm{RS}+u_{0j} \end{array}$$

In step-3, a model was tested by adding level-1 explanatory variables into the random intercept and slop logit model. The multilevel logit model was as follows:

$$\begin{array}{cc} \;\log\left(\frac{p_{\mathrm{ij}}}{1-p_{\mathrm{ij}}}\right) & =\beta_{0j}+\beta_{0j}DD_{\mathrm{ij}}+\beta_{2}\mathrm{Gende}r_{\mathrm{ij}}\\ & +\beta_{3}\mathrm{Employmen}t_{\mathrm{ij}}+\beta_{4}\mathrm{PCH}I_{\mathrm{ij}}\\ & +\beta_{5}PS_{\mathrm{ij}}\beta_{0j}=\gamma_{00}+\gamma_{01}\mathrm{PCI}V_{j}\\ & +\gamma_{02}\mathrm{NP}C_{j}+\gamma_{03}\mathrm{SC}D_{j}+\gamma_{04}RS_{j}\\ & +u_{0j}\beta_{1j}=\gamma_{10}+\gamma_{11}\mathrm{PCI}V_{j}+u_{1j} \end{array}$$

The combined model was:

$$\begin{array}{ll} \;\log\left(\frac{p_{\mathrm{ij}}}{1-p_{\mathrm{ij}}}\right) & =\gamma_{00}+\gamma_{01}PCIV_{j}+\gamma_{02}NPC_{j}+\gamma_{03}SCD_{j}\\ & +\gamma_{04}RS_{j}+\gamma_{10}DD_{\mathrm{ij}}+\gamma_{11}PCIV_{j}*DD_{\mathrm{ij}}\\ & +\beta_{2}{\text{Gender}}_{\mathrm{ij}}+\beta_{3}{\text{Employment}}_{\mathrm{ij}}\\ & +\beta_{4}PCHI_{\mathrm{ij}}+\beta_{5}PS_{\mathrm{ij}}+(u_{0j}+u_{1j}*DD_{\mathrm{ij}}) \end{array}$$

The glimmixed and nlmixed procedures of the SAS9.13 were used for statistical analysis [20].

## Results

### Descriptive analysis

In this study, the total number of the disabled individuals with rural residence over the age of 18 was 114,485. Of those, 57,296 were male (50%) and 57,189 were female (50%). As such, the other individual-level and community-level characteristics of the participants are shown in Table 1.

**Table 1:** Descriptive statistics of rural disabled persons’ independent variables and social medical insurance participation rate and significance of univariate analysis

**Variable**	**Sample size and proportion N (%)**	**Social medical insurance participation rate (%)**
Participation in social medical insurance		
Yes	32374 (28)	
No	82111 (72)	
	**Level 2 characteristic N=5220**	
Rehabilitation Stations**		
Yes	1014 (19)	36
No	4206 (81)	27
Special Committees for the Disabled**		
Yes	2369 (45)	30
No	2851 (55)	27
NPC**		
Yes	750 (14)	41
No	4470 (86)	26
Per Capita Income of Villagers in 2005**		
683 or less	1157 (22)	19
684–2800	2083 (40)	24
2301 or more	1980 (38)	35
	**Level 1 characteristic N=114,485**	
Gender*		
Male	57296 (50)	29
Female	57189 (50)	28
Educational Level**		
College or Above	109 (0)	41
Primary, High and Technical Secondary School	16785 (15)	30
Primary School and Below	97591 (85)	28
Employment**		
Yes	41521 (36)	29
No	72964 (64)	28
Marital Status**		
In Marriage	69340 (61)	29
Not in Marriage	45145 (39)	27
Per Capita Household Income(PCHI)**	2281.32 (STD=2264.54)	
683 or less	15117 (13)	21
684–2300	59726 (52)	27
2301 or more	39642 (35)	33
Disability Degree**		
grade-1 (most severe)	20030 (18)	27
grade-2	14086 (12)	26
grade-3	30947 (27)	28
grade-4 (least severe)	49422 (43)	30
Participation in Society**		
None/ Mild barriers	51328 (45)	29
Moderate barriers	36343 (32)	29
Severe barriers	26814 (23)	26

*Significant at 0.05,

**Significant at 0.001, significance of the *x*^*2*^*-test*, examining the independent associations between rural disabled persons’ social medical insurance participation and independent variables.

### Univariate analysis

Among the rural disabled persons investigated in 2006, only 32,374 participated in social medical insurance, accounting for 28%. Compared with the disabled rural individuals who were unemployed, with lower PCHI, with more severe disability degree or with more severe barriers in participation in society respectively, those employed with higher PCHI, with less severe disability degree or with less severe barriers in participation in society had significantly higher social medical insurance participation rates (p < 0.001). As for the community-level variables, the rural disabled persons in the area with rehabilitation stations, with SCD, in NPC or with a higher PCIV had significantly higher social medical insurance participation rates than those in the area without rehabilitation stations, without SCD, in non-NPC or with lower PCIV (p < 0.001, Table 1)

### Multilevel analysis

The analysis of the models verified the influence of policy and economic factors on rural disabled persons’ participation in social medical insurance and further showed the relevance between rural disabled persons’ disability degree and their participation in social medical insurance. However, the interaction between the policy and economic factors and the disability degree was not statistically significant. Therefore, it was uncertain whether the influence of contextual factors on rural disabled persons’ participation in social medical insurance would vary with the disability degree. The detailed steps were as follows.

A null model was developed to assess the within-group homogeneity, which turned out to be quite large (See Statistical analysis). The community-level variables were added to model 1. The results showed that the odds of participating in social medical insurance of rural disabled persons in the area with RS, in NPC and with higher PCIV were higher, with statistical significance. However, SCD did not influence rural disabled persons’ participation in social medical insurance. The individual-level variables including disability degree, participation in society, PCHI and employment were added to model 2. All the results were statistically significant with a −2*Log Likelihood (LL) of 658,390.6. Therefore, the odds of participating in social medical insurance of rural disabled persons with more severe disability degree, more severe barriers in participation in society, lower PCHI or no job were lower.

The individual-level background variables such as gender and age were added to model 3 as covariates. The results showed that the age was not statistically significant, thus it was removed from the analysis. Finally, the -2LL of model 3 was 658,366.3. The difference in -2LL (likelihood ratio (LR) = 24.3, df = 2, p < 0.0001) between model 3 and 2 indicated that the fit of model 3 improved over model 2 with an acceptable statistical significance. In order to discuss how NRCMS pilot policy mediated the effects of disability degree on rural disabled persons’ social medical insurance participation, the cross-level interaction between NPC and disability degree was added to model 4. The results showed that the interaction was not statistically significant with a -2LL of 658,387.4 for model 4. The difference in -2LL (LR = −21.1, df = 2, p < 0.0001) between model 4 and 3 indicated that the fit of model 3 was better. Meanwhile, the cross-level interaction between disability degree, PCHI, participation in society, employment and other community-level variables were also tested but showed no statistical significance. Therefore, model 3 was chosen in the end.

Based on the results gathered from the assessment of model 3, the contextual factors including policy and economic contexts had important influence on rural disabled persons’ participation in social medical insurance in China. A statistically significant relationship was found in 3 community-level variables. More specifically, rural disabled persons in NPC had higher odds of participating in social medical insurance as compared to those in non-NPC (odds ratio [OR]: 3.34; 95% confidence interval [CI]: 2.62-4.25). Furthermore, rural disabled persons in the area with rehabilitation stations had higher odds of participating in social medical insurance than those without rehabilitation stations (OR: 1.87; 95% CI: 1.49-2.36). Rural disabled persons with higher PCIV were more likely to participate in social medical insurance. The rural disabled persons with PCIV of less than 683 Yuan or even l,684 to 2,800 Yuan had lower odds of participation in social medical insurance than those with more than 2,801 Yuan (OR: 0.36; 95% CI: 0.28-0.46 and OR: 0.45; 95% CI: 0.37-0.55, Table 2).

**Table 2:** Estimates and test results of model 3

**Odd ratio (95% CI)**				
**Variable**	**Model 1**	**Model 2**	**Model 3**	**Model 4**
**Level-2**				
Special Committees for Disability				
Yes	1.00 (0.82, 1.21)	0.98 (0.81, 1.20)		
No	1.0	1.0		
Rehabilitation Station				
Yes	1.91 (1.49, 2.45) **	1.88 (1.47, 2.41) **	1.87 (1.49, 2.36) **	1.87 (1.49, 2.36) **
No	1.0	1.0	1.0	1.0
NPC				
Yes	3.36 (2.64, 4.27) **	3.34 (2.62, 4.25) **	3.34 (2.62, 4.25) **	3.38 (2.65, 4.31) **
No	1.0	1.0	1.0	1.0
Per Capita Income of Villagers in 2005				
683 or less	0.37 (0.29, 0.48) **	0.36 (0.28, 0.46) **	0.36 (0.28, 0.46) **	0.36 (0.28, 0.46) **
684–2800	0.43 (0.35, 0.52) **	0.45 (0.37, 0.55) **	0.45 (0.37, 0.55) **	0.45 (0.37, 0.55) **
2301 or more	1.0	1.0	1.0	1.0
**Level-1**				
Intercept	0.15 (0.12, 0.18)**	0.096 (0.08, 0.11)**	0.098 (0.09, 0.11)**	0.16 (0.14, 0.19)**
Gender				
Male			1.07 (1.02, 1.11) **	1.07 (1.02, 1.11) **
Female			1.0	1.0
Employment				
Yes		1.12 (1.07, 1.18) **	1.11 (1.06, 1.16) **	1.11 (1.06, 1.16) **
No		1.0	1.0	1.0
Per Capita Household Income in 2005				
683 or less		0.69 (0.64, 0.75) **	0.68 (0.63, 0.74) **	0.69 (0.63, 0.74) **
684–2300		0.84 (0.80, 0.88) **	0.83 (0.79, 0.88) **	0.83 (0.79, 0.88) **
2301 or more		1.0	1.0	1.0
Disability Degree				
grade-1 (most severe)		0.80 (0.75, 0.86) **	0.80 (0.75, 0.86) **	0.82 (0.75, 0.89) **
grade-2		0.81 (0.75, 0.87) **	0.80 (0.75, 0.87) **	0.82 (0.74, 0.90) **
grade-3		0.82 (0.78, 0.86) **	0.82 (0.77, 0.86) **	0.80 (0.75, 0.86) **
grade-4 (least severe)		1.0	1.0	1.0
Participation in Society				
None/ Mild barriers		1.10 (1.02, 1.18) *	1.10 (1.03, 1.18) *	1.10 (1.03, 1.18) *
Moderate barriers		1.10 (1.03, 1.18) *	1.10 (1.03, 1.18) *	1.10 (1.03, 1.18) *
Severe barriers		1.0	1.0	1.0
**Cross-Level Interactions**				
social medical insurance Pilot County & Disability Degree				
Yes& grade-1				1.06 (0.91, 1.24)
Yes&grade-2				1.04 (0.87, 1.26)
Yes&grade-3				0.95 (0.83, 1.09)
Yes&grade-4				1.0

* Significant at 0.05,

** Significant at 0.001, significance of the two-level logistic regression analysis, assessing the association between independent variables and rural disabled persons’ social medical insurance participation.

Other important influencing factors were employment, disability degree and participation in society. Rural disabled persons who were employed had higher odds of participating in social medical insurance as compared to those unemployed (OR: 1.11; 95% CI: 1.06-1.16). As for the disability degree, rural disabled persons with more severe disability were less likely to participate in social medical insurance. Rural disabled persons with a grade-1, 2 or 3 disability had lower odds of participating in social medical insurance as compared to those with a grade-4 disability (OR: 0.80, 95% CI: 0.75-0.86; OR: 0.80, 95% CI: 0.75-0.87; OR: 0.82, 95% CI: 0.77-0.86, respectively). Rural disabled persons with no barrier, mild or moderate barriers in participation in society had higher odds of participating in social medical insurance as compared to those with severe barriers (OR: 1.10; 95% CI: 1.03-1.18 and OR: 1.10; 95% CI: 1.03-1.18, respectively, Table 2).

## Discussion

The analysis above showed that the contextual factors including policy and economic contexts were important factors influencing rural disabled persons’ participating in social medical insurance in China and unemployment. Furthermore, severer disability degree and social isolation might also deeply violate their equal access to social medical insurance. In China, policy is the most important factor influencing the rural disabled persons’ participation in social medical insurance. Our results showed that the odds of participating in social medical insurance of rural disabled persons in NPC were 3.34 times larger as compared to those in non-NPC, which also applies to the real situation.

At the end of 2003, China began to launch NRCMS pilots in part of China [21], which amounted to about 40% of the national counties (cities and districts) in 2006 [22]. The social medical insurance participation rates in various pilot studies have been on the rise since 2003 with the spread of NRCMS pilots. Wusheng county, the NPC in Sichuang province since August 2003, had a total of 1,311,000 person-times participating in social medical insurance over the past three years, of which 435,100 participated from 2003 to 2004 (participation rate=61%), 371,000 (51%) in 2005 and 504,900 (69%) in 2006 [23]. Meanwhile, the low self-paid premium of NRCMS largely promoted rural disabled persons’ participation in social medical insurance. In 2003, the minimum annual self-paid premium of NRCMS was only 10 Yuan while the corresponding average payment of the medical insurance for urban workers was 271 Yuan [24]. Therefore, the policy was the most important factor influencing rural disabled persons’ participation in social medical insurance. However, there was no NRCMS related central policy specified for rural disabled persons, which might influence rural disabled persons’ participation in social medical insurance, especially those with lower PCHI or more severe disability degree.

These results showed that the odds of participating in social medical insurance of rural disabled persons in the area with rehabilitation stations were 1.87 times larger than those without rehabilitation stations. In 2002, “Views on Further Strengthening Rehabilitation Work for Disabled Persons” [25] pointed out the importance of introducing rehabilitation work into the community for disabled persons by gradually providing the community and household rehabilitation services to the disabled persons in order to raise funds through multiple channels for the rehabilitation aid of poor disabled persons, to construct the rehabilitation infrastructure for disabled persons and to carry out post-rehabilitation professional and labor skills to promote education, employment and full participation in society for disabled persons. The establishment of rehabilitation stations might reflect the enforcement of rehabilitation policy. Strict enforcement of this policy can largely promote rehabilitation services, skill training, education and employment for disabled persons, which could increase the probability of their participation in social medical insurance.

These results showed that the rural disabled persons with higher PCHI or in the area with higher PCIV were more likely to participate in medical insurance. The areas with better economic status might provide more preferential policies for the rural disabled, such as lower self-paid premium and higher hospitalization reimbursement. Our results showed that the rural disabled persons in the areas with better economic status had higher initiatives to participate in social medical insurance. Although the minimum self-paid premium of NRCMS was only 10 Yuan, the rural disabled persons with low income were more likely to spend the money on consumer goods to obtain maximum utility. The satisfaction survey of NRCMS in Liaoning province showed that the farmers with a better economic status or better health status showed higher satisfaction with NRCMS [26]. Thus rural disabled persons with worse economic status or worse health status benefited less from NRCMS. Furthermore, due to the low financing level, low reimbursement rate and limited disease coverage, without corresponding preferential policies, the rural disabled with poor economic status had low initiatives to participate in social medical insurance.

The odds of participating in social medical insurance of employed rural disabled persons were 1.11 times higher than that of the unemployed. In 2006, the social medical insurance in China was mainly consisted of medical insurance for urban workers, which had already been spread all over China, and NRCMS, which only piloted in a part of China. Medical insurance for urban workers covered all the workers of townships and village enterprises. Therefore, the employed rural disabled persons were covered by medical insurance for urban workers and obtained medical security.

As for the disability degree and participation in society, rural disabled persons with more severe disability or barriers were less likely to participate in social medical insurance. The disability reduced the probability of being employed, especially for those with severe disability, thus reduced the odds of participation in social medical insurance. Also, disability and unemployment worsened the economic status and restrained their knowledge of policy due to the barriers thus reduced the odds of participation in social medical insurance. Based on these findings, we concluded that China rural disabled persons’ medical security was constrained by their disabilities and social participation barriers before 2006, which might have seriously prevented them from gaining equal access to medical security.

## Conclusions

The analysis above showed that contextual factors, including economic and policy contexts, were important factors influencing rural disabled persons’ participation in social medical insurance before 2006 in China. Unemployment, severer disability degree and social isolation might also largely prevent them from gaining equal access to social medical insurance. Therefore, the key for disabled persons to obtain equal medical security is to develop comprehensive social medical insurance policies and a series of strategies ensuring the strict implementation. Employment and education opportunities should be created to help disabled persons get back to normal life and obtain social medical insurance. Rehabilitation services should be strengthened to help disabled persons regain or compensate functions, improve quality of life and enhance social participation. With these measures, the disabled persons’ rights of equal access to medical security could be guaranteed and their human rights and social civilization could also be promoted. Just as the *Convention on the Rights of Persons with Disabilities* said, “to promote, protect and guarantee sufficient and equal access to all human rights and fundamental freedoms of the disabled, and promote respect for the inherent dignity of the disabled” [2].

### Limitations

There were some limitations in this study. The SCNSSD in 2006 was designed to provide reference data for the disability cause, so the survey data on social medical insurance might have not been comprehensive and might have affected the construction of the proposed model. However, this data met the need for studying the influencing factors of social medical insurance for the disabled with high quality and can be used. Furthermore, this study had a huge data volume and multiple variables, which largely slowed the operational speed of running multilevel model and resulted in non-convergent results. Therefore, in the model operation, we tried to simplify the model to improve the operational efficiency.

## Endnotes

^a^ According to *Convention on the Rights of Person with Disabilities*[2], disability is an evolving concept and disability results from the interaction between persons with impairments and attitudinal and environmental barriers that hinders their full and effective participation in society on an equal basis with others. Persons with disabilities include those who have long-term physical, mental, intellectual or sensory impairments which in interaction with various barriers may hinder their full and effective participation in society on an equal basis with others. In the context of the International Classification of Functioning, Disability and Health [27], disability is an umbrella term, covering impairments, activity limitations and participation restrictions. An impairment is a problem in body function or structure; an activity limitation is a difficulty encountered by an individual in executing a task or action; while a participation restriction is a problem experienced by an individual in involvement in life situations. Thus disability is a complex phenomenon, reflecting an interaction between features of a person’s body and features of the society in which he or she lives.

## Abbreviations

ICC: Intraclass Correlation Coefficient;NPC: NRCMS Pilot County;NRCMS: New Rural Cooperative Medical System;PCHI: Per Capita Households Income;PCIV: Per Capita Income of the Villagers in 2005;SCD: Special Committees for the Disabled;SCNSSD: Second China National Sample Survey on Disability

## Competing interests

This study was funded by China Disabled Persons’ Federation (Project Number: 2010&YB004). In 2010, China Disabled Persons’ Federation granted us an amount of funding, which was managed by Peking University Health Science Center. Since the funding was limited, it could not cover the costs of this manuscript. Therefore, the authors had to pay a proportion of the cost. The authors declared that they had no other financial competing interests.

## Authors’ contributions

This paper took the jointed efforts of the three authors. JH conceived of the original idea and participated in its data collection, statistical analysis and writing. XLP participated in the study design, critical revision and coordination. AL participated in the statistical analysis, writing, revision and translation. All authors read and approved the final manuscript.

## Pre-publication history

The pre-publication history for this paper can be accessed here:

http://www.biomedcentral.com/1472-6963/13/58/prepub
